# To: Comparison of bronchial hygiene techniques in mechanically ventilated patients: a randomized clinical trial

**DOI:** 10.5935/0103-507X.20190080

**Published:** 2019

**Authors:** Angelo Roncalli Miranda Rocha, George Ntoumenopoulos, Luiz Alberto Forgiarini Júnior

**Affiliations:** 1 Departamento de Fisioterapia, Centro Universitário CESMAC - Maceió (AL), Brasil.; 2 Saint Vincent's Hospital Sydney - Darlinghurst, New South Wales, Austrália.; 3 Programa de Pós-Graduação em Saúde e Desenvolvimento Humano e Curso de Fisioterapia, Universidade La Salle - Canoas (RS), Brasil.

**To the Editor**

In this issue of *Revista Brasileira de Terapia Intensiva* , Naue et al.^(1)^ present an interesting study. We would like to congratulate the authors for their relevant contribution to the scientific literature on the subject, but we need clarification on the study design and reporting.

The results of the study show that hyperinflation with a mechanical ventilator (HMV)+vibrocompression (VB) increased the amount of aspirated secretions, which did not occur with the other techniques. However, when HMV was applied alone and compared to pulmonary aspiration alone (ASP), the amount of secretion removed did not differ significantly, and worse, according to figure 3, it appears that ASP was able to remove more secretions than HMV, although there was no statistical significance. This led us to reflect on the reason why HMV, particularly the way it was performed, could have yielded the opposite effect to that proposed; that is, why did it displace the secretion towards the periphery of the lungs? In the protocol used, HMV was performed in a pressure-controlled mode (which the authors erroneously classified as a "pressure cycling mode"), with inspiratory pressure increasing until a peak pressure of 40cmH_2_O was reached. We believe that the use of pressure-controlled modes does not favor a peak expiratory flow 10% higher than the peak inspiratory flow, which is necessary for the displacement of mucus towards the glottis by two-phase liquid-gas interaction (expiratory flow bias).^(2)^

Thomas^(3)^ demonstrated in a bench model that a ventilation mode with volume control/flow is more successful in achieving an expiratory flow bias, which is theoretically necessary for adequate clearance of the pulmonary secretions. This is due to the ability to control peak inspiratory flow, which is not possible in pressure-controlled modes: in these, the inspiratory flow is variable and adapts according to respiratory system compliance, airway resistance and patient inspiratory effort. In a randomized crossover study, Amaral et al.^(4)^ observed that the expiratory flow bias was significantly greater in volume-controlled mode than in pressure-controlled mode. By increasing the inspiratory pressure, the authors demonstrated an increase in inspiratory flow in a ratio at least proportional to the expiratory flow, reducing the chances of obtaining a PIF/PEF ratio ≤ 0.9, which is necessary to move the mucus in the direction to the glottis. Our question is: if the HMV had been performed in volume-controlled mode, could there have been more effective secretion clearance?^(2)^

The authors also did not standardize the inspiratory rise time. This parameter, when adjusted for the same inspiratory pressure level, can alter the inspiratory flow to be larger (if the rise/pressurize time is faster) or smaller (shorter rise/pressurize time). The lack of a standardized adjustment may have caused different PIF/PEF relationships for the same time and inspiratory pressure adjustment.^(3,4)^ We would also argue that mechanical ventilation settings are not the only means of enhancing secretion clearance; large animal model studies demonstrate the benefit of the manual chest wall compression technique (harder compression and early timing)^(5)^ and gravity (head-down).^(6)^

Finally, the authors incorrectly related the short-term interventions with the longitudinal outcomes for ventilation duration and mortality. We fail to understand how a randomized crossover trial with a single intervention can determine outcomes from longitudinal studies, as if patients had received the same intervention throughout their intensive care unit stay.

## References

[1] Naue WS, Herve BB, Vieira FN, Deponti GN, Martins LF, Dias AS (2019). Comparison of bronchial hygiene techniques in mechanically ventilated patients: a randomized clinical trial. Rev Bras Ter Intensiva.

[2] Volpe MS, Adams AB, Amato MB, Marini JJ (2008). Ventilation patterns influence airway secretion movement. Respir Care.

[3] Thomas PJ (2015). The effect of mechanical ventilator settings during ventilator hyperinflation techniques: a bench-top analysis. Anaesth Intensive Care.

[4] Amaral BL, de Figueiredo AB, Lorena DM, Oliveira AC, Carvalho NC, Volpe MS (2019). Effects of ventilation mode and manual chest compression on flow bias during the positive end- and zero end-expiratory pressure manoeuvre in mechanically ventilated patients: a randomized crossover trial. Physiotherapy.

[5] Ntoumenopoulos G, Shannon H, Main E (2011). Do commonly used ventilator settings for mechanically ventilated adults have the potential to embed secretions or promote clearance?. Respir Care.

[6] Marti JD, Li Bassi G, Rigol M, Saucedo L, Ranzani OT, Esperatti M (2013). Effects of manual rib cage compressions on expiratory flow and mucus clearance during mechanical ventilation. Crit Care Med.

